# The Impact of Internet Medical Information Overflow on Residents’ Medical Expenditure Based on China’s Observations

**DOI:** 10.3390/ijerph17103539

**Published:** 2020-05-19

**Authors:** Junqiang Han, Xiaodong Zhang, Yingying Meng

**Affiliations:** 1 School of Public Management, South-Central University for Nationalities, Wuhan 430074, China; junqianghan@scuec.edu.cn; 2 Centre for Social Security Studies, Wuhan University, Wuhan 430072, China

**Keywords:** Internet use, medical expenditure, information asymmetry, China

## Abstract

*Background*: The rapid rise of medical expenditure is a common problem in the field of public health around the world, but the challenges for the Chinese government are even greater. How to control the rapid rise in medical expenditure and reduce individuals’ economic burden when receiving medical treatment has become one of the core issues that the Chinese government urgently needs to solve. The aim of this study was to evaluate the impact of Internet use on individuals’ medical expenditure and further discuss the potential impact mechanism. *Methods*: The data used in this study were from the 2018 China Family Panel Studies (CFPS) conducted by Peking University. The Heckman sample selection model was used to analyse the impact of Internet use on individuals’ medical expenditure. *Results*: Internet use reduced the medical expenditure of individuals by 6.19%; high frequency Internet use reduced the medical expenditure of individuals by 15.1%, while low frequency Internet use had no impact. In addition, Internet use had different impacts on individuals’ medical expenditure at different levels of hospitals. Specifically, Internet use reduced the medical expenditure of individuals who received medical treatment at general hospitals by 9.63%, and high frequency Internet use reduced the medical expenditure of individuals by 22.2%. However, Internet use had no impact on the medical expenditure of individuals who received medical treatment at primary hospitals. *Conclusions*: Findings from this study underscore the importance of Internet use as an important role in reducing individuals’ medical expenditure. The use of the Internet can significantly reduce the level of individuals’ medical expenditure, and high frequency Internet use has a greater effect. However, Internet use has different impacts on individuals’ medical expenditure among different levels of hospitals. The reduction effect of Internet use on individuals’ medical expenditure is mainly concentrated in general hospitals but has no effect in primary hospitals.

## 1. Introduction

**Figure A1:**
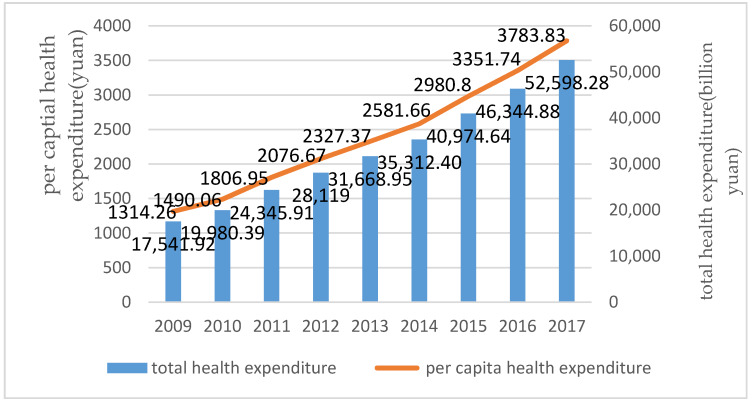
Changes of total health expenditure and per capital health expenditure.

The rapid rise of health care costs is a common problem in the field of public health around the world, but the challenges for Chinese government have been even greater in recent years. The Chinese government launched a new round of medical and health system reform, in 2009, to comprehensively improve the health status of residents and reduce the financial burden of medical treatment, and one of the main initiatives was to massively increase the financial funds in the field of public health. According to data released by the National Bureau of Statistics, from 2009 to 2017, the government expenditure on health care increased from 481.626 billion RMB to 1520.587 billion RMB, an increase of 3.16 times. However, the outcome of the reform appears to have been contrary to expectations, i.e., from 2009 to 2017, China’s total health expenditure increased from 17,541.92 billion RMB to 52,598.28 billion RMB, with an average annual growth rate of 14.75%; and the per capita health expenditure increased from 1314.26 RMB to 3783.83 RMB (see Figure A1), with an average annual growth rate of 14.17%. It is obvious that the rapid growth of medical expenditure has become one of the major challenges that the Chinese government faces in medical and health reform.

Regarding the factors that influence the rapid growth of residents’ health expenditure, the existing literature has mainly been analysed from the macro and micro perspectives. From a macro perspective, scholars believe that technological progress, demographic changes, the arrival of an ageing society, economic growth, urbanization, health insurance coverage, rapid development of the medical industry, and defects in the medical management system are the reasons for the sharp rise in residents’ health costs [1,2,3,4,5,6,7,8,9,10,11]. From a micro perspective, individual and family income growth, people’s health demand, the third-party payment mechanism that comes with participating in the medical insurance system, and other factors are considered to be the personal reasons for the rapid increase in residents’ health expenditure [2,12,13,14]. Among these factors, the phenomenon of “excessive medical treatment” is particularly noticeable in the medical service market, due to information asymmetry between doctors and patients that further leads to the rise of residents’ health care costs.

In 1963, Arrow introduced “uncertainty” into the public health field and concluded that the risk of uncertainty in disease occurrence and medical treatment induced information asymmetry between doctors and patients [15,16]. On the basis of Arrow’s study, many scholars found that under information asymmetry, doctors have an incentive to “induce” patients to “excessively” consume medical services in order to obtain more benefits, and this kind of incentive leads to the rapid rise of health care costs for residents [1,7,17]. At the same time, this “induction effect” is also affected by the supply of medical information. Patients can effectively reduce the “excessive medical treatment” and further reduce the medical expenditure by seeking more medical information [18,19]. Therefore, how to reduce the information asymmetry and increase the supply of health information has become one key step to control for the excessive growth of medical expenditure.

With the advance of Internet technology, searching for health information online is becoming more and more popular among the general population [20], and this could reduce the information asymmetry to some extent. Numerous studies have suggested that various types of health information obtained through the Internet play an important role in individuals’ self-diagnosis of disease [21,22], self-treatment of chronic disease [23], doctor-patient relationship regulation [24], and many other behaviors. Some studies have suggested that many patients search for health information through the Internet and use this information to decide whether or not to visit a doctor [25,26]. Several scholars even found high-quality online health information can be effective to improve self-management and reduce healthcare usage in times of increasing healthcare costs [27]. 

In reality, this paper holds that Internet use can reduce information asymmetry between doctors and patients in the field of medical services. Previous studies have found that Internet use broadens the means for the dissemination and acquisition of medical information, making the concept of “medical information” transition from static knowledge to dynamic information overflow. The structural change of medical information supply is likely to affect patients’ treatment behavior. Patients who have access to more professional medical knowledge through the Internet, which allows them to better understand their own health status and choose rational medical resources, and also helps to eliminate the professional knowledge barriers in doctor-patient communication, can more actively participate in the treatment decision-making process [28,29]. 

**Figure A2:**
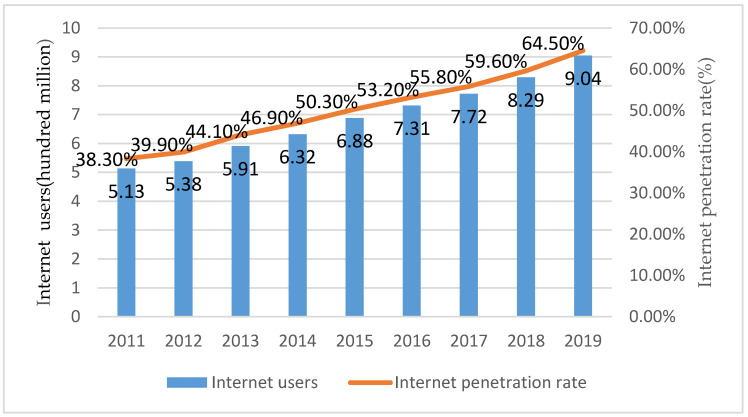
Changes of Internet users and Internet penetration rate.

After reviewing the relevant literature, we found that previous studies have not paid enough attention to the impact of Internet technology on residents’ medical expenditure, based on the reality that medical expenditure is increasing rapidly. To date, China has entered the age of the Internet. According to the report released by the China Internet Information Centre, from 2011 to 2019, the number of China’s Internet users increased from 513 million to 904 million and the Internet penetration rate increased from 38.3% to 64.5% (see Figure A2). In the information age of the Internet, it has become very common for residents to master health-related information through the Internet [8,30]. For this paper, we used the 2018 China Family Panel Studies (CFPS) data to explore the relationship between Internet use and individuals’ medical costs in the context of informatization, and we further discussed the potential impact mechanism.

## 2. Data and Methods 

### 2.1. Data

The data used for this paper were retrieved from the 2018 CFPS survey, which aimed to reflect the changes among Chinese families by collecting data at three levels, i.e., individuals, families, and communities. The CFPS survey covers various research topics, including family finances, marital status, social security, health care costs, health status, Internet use, and so on. The survey encompassed the country’s 25 provinces/cities/autonomous regions and adopted a multistage, implicit stratification and population-scale method (PPS) that integrated urban and rural areas. The population of the sample collection area accounts for 94.5% of the country’s total population. The data are currently representative of large-scale micro-integrated social survey data in China.

The 2018 CFPS database consists of a family database, an adult database, and a children’s database. Due to research needs, we used data from the adult database and the family database for the study analysis. First, we matched the data from the adult database with those of the family database and deleted the duplicate samples to obtain a sample size of 32,669. Second, we removed the samples with responses to core variables of “I don’t know”, “I refuse to answer”, or “not applicable”, as well as those with missing values, and thus a final sample size of 27,890 was used to analyse the impact of Internet use on individuals’ health care costs.

### 2.2. Variables’ Measurement

#### 2.2.1. Outcome Variable

The outcome variable of this study was the amount of individual’s health care costs. According to the design of the 2018 CFPS questionnaire, we chose the following question to measure the individuals’ medical expenditure: “In the past year, how much money (including the amount reimbursed or to be reimbursed) has been spent on your medical care (including medicine, medical treatment, hospitalization, etc.)?” Table 1 shows that the average medical expenditure of respondents was 4.9 yuan (log) in the past year.

#### 2.2.2. Explanatory Variable

The core explanatory variable of this paper was the Internet use status of Chinese residents. We chose two indicators, namely, Internet access and the frequency of Internet use, to measure the Internet use status of respondents. First, we chose the following question from the 2018 CFPS questionnaire to measure the Internet access of respondents: “Do you use a computer to access the Internet?” The respondents were assigned a value of 1 if they chose “Yes”, or a value of 0 otherwise. Second, we chose the question “In general, how frequently do you use the Internet?” to measure the frequency of Internet use, and the answer choices were as follows: (1) every day, (2) 3–4 times per week, (3) 1–2 times per week, (4) 2–3 times per month, (5) once per month, (6) once every few months, and (7) never. In the process of our empirical analysis, we generated three dummy variables, namely, “never”, “sometimes” and “almost every day”. Specifically, when respondents chose “never”, they were assigned a value of 1, or a value of 0 otherwise; when they chose “1–2 times per week”, “2–3 times per month”, “once per month”, or "once every few months”, they were assigned a value of 1, or 0 otherwise; when they chose “every day” or “3–4 times per week”, they were assigned a value of 1, or 0 otherwise. Table 1 shows that 8.63% of individuals used the Internet almost every day, 40.2% of individuals used the Internet sometimes, and 51.1% of individuals never utilized the Internet.

#### 2.2.3. Controlled Variables 

To reduce possible bias in the statistical model due to omitted variables, four types of variables were controlled for in the empirical analysis: (1) individual characteristics, including age, gender, marriage status, *hukou* (Hukou is household register registration system, which consists of rural household registration and urban household registration in China.), religious belief, and family size; (2) health status, including self-rated health, chronic disease, smoking, drinking, and the frequency of exercise per week; and (3) socioeconomic status indicators, including education level, annual household income, health insurance, and hospital level at which the individuals receive medical treatment; (4) meanwhile, considering that differences in the economic development level and Internet coverage among different provinces and cities in China can have an impact on the results, in the empirical analysis, we controlled for the provinces where the individuals were located. The specific definitions are presented in Table 1.

### 2.3. Heckman Sample Selection Model 

While processing the data, we found that some individuals had not generated a medical expenditure in the past year. If individuals chose not to receive medical treatment after being ill, we could not observe the real medical expenditure of these samples, which would result in sample selection bias. Therefore, this study used the Heckman sample selection model to solve the possible selective deviation in the sample.

The Heckman sample selection model consists of a selection equation and an expenditure equation. The selection equation is mainly used to estimate the probability of receiving medical treatment and is estimated by using the probit model, which is set as follows:

$$Prob\left(Z_{i}=1|W_{i}\right)=\beta_{0}+\overset{k}{\underset{i=1}{\sum }}\beta_{i}W_{i}$$

where $Prob\left(Z_{i}=1|W_{i}\right)$ represents the probability of receiving medical treatment and $Z_{i}$ is the dependent variable in the first stage ($Z_{i}$ = 1 represents the *i*-th individual who received medical treatment and $Z_{i\;}$= 0 represents the *i*-th individual who did not receive medical treatment); $\beta_{0}$ is the intercept term; $\overset{k}{\underset{i=1}{\sum }}Wi$ represents a series of controlled variables, including individual characteristics, living habits, health status, and socioeconomic indicators; $\beta_{i}$ indicates the coefficient of the impact of these controlled variables on the respondent’s medical treatment; and ε is a random error term.

The second stage is the individual’s medical expenditure equation, mainly examining the impact of Internet use on the individual’s medical expenditure, which is the focus of this paper. The model is set as follows:

$$LnExpenditure=\beta_{0}+\beta_{1}Internet_{i}+\beta_{i}\overset{k}{\underset{i=1}{\sum }}Wi+\varepsilon$$

where LnExpenditure represents the medical expenditure of the respondents and $\beta_{0}$ is the intercept term; $Internet_{i}$ represents the Internet use status of the individual; $\beta_{1}$ indicates the coefficient of the impact of Internet use on the respondent’s medical expenditure; $\overset{k}{\underset{i=1}{\sum }}Wi$ represents a series of controlled variables, including individual characteristics, living habits, health status, and socioeconomic indicators; $\beta_{i}$ indicates the coefficient of the impact of these controlled variables on the respondent’s medical expenditure; and ε is a random error term. 

## 3. Results

Table 2 reports the regression results of the impact of Internet use on individuals’ medical expenditure. Columns (1)–(3) add individual characteristic variables, health status and socioeconomic indicators, in order, and report the estimation results of the impact of Internet use on individuals’ medical expenditure. The regression results suggest that, after controlling for the individual characteristic variables, health status and socioeconomic indictors, Internet use can reduce individuals’ medical expenditure by 6.19% as compared with individuals who never use the Internet, and this result was significant at the 10% statistical level, which implies that Internet use can reduce individuals’ medical expenditure.

Columns (4)–(6) add individual characteristic variables, health status, and socioeconomic indicators, in order, and report the estimation results of the impact of Internet use frequency on individuals’ medical expenditure. We categorized Internet use frequency into “almost every day” (high frequency), “sometimes” (low frequency), and “never”. The baseline variable was “never”. The regression results show that after controlling for the individual characteristic variables, health status and socioeconomic indictors, utilizing the Internet almost every day can reduce medical expenditure by 15.1% as compared with individuals who never use the Internet, and this result was significant at the 1% statistical level, which means that high frequency Internet use can significantly reduce individuals’ medical expenditure. However, utilizing the Internet sometimes has no impact on individuals’ medical expenditure, which implies that low frequency Internet use has no effect on individuals’ medical spending.

It is worth noting that there are large gaps in health resources distribution in China. China’s medical and health resources are mainly concentrated in general hospitals (including general hospitals and specialized hospitals) and relatively scarce in primary hospitals (including community health service center/health clinic in town or township, community health station/village clinic and private clinic). High-tech, advanced equipment, and excellent experts in the field of health services are basically concentrated in general hospitals in cities, attracting crowds of patients. The rapid rise of medical expenditure has mainly occurred in general hospitals, where the doctors have the incentive to induce demand and generate excessive medical treatment caused by information asymmetry. On the contrary, the growth space of medical expenditure is limited in primary hospitals, so theoretically, the impact of Internet use on the medical expenditure varies among different hospital levels. 

Table 3 divides the hospital levels into general hospitals and primary hospitals and further reports the impact of Internet use on individuals’ medical expenditure among different hospital levels. Specifically, Column (1) in Table 3 reports the impact of Internet use on the medical expenditure of individuals treated in general hospitals. The results of the regression analysis suggested that, under the same conditions, Internet use reduced the medical expenditure of individuals treated in general hospitals by 9.63%, and this result was significant at the level of 10%. Column (2) reports the impact of Internet use on the medical expenditure of individuals treated in primary hospitals. The regression analysis results showed that Internet use had no impact on the medical expenditure of individuals treated in primary hospitals.

Column (3) in Table 3 further reports the impact of the frequency of Internet use on the medical expenditure of individuals treated in general hospitals. We divided the Internet use frequency into “almost every day”, “sometimes”, and “never”, and the baseline variable was “never”. Results of the regression analysis showed that under the same conditions as compared with individuals who never used the Internet, utilizing the Internet almost every day reduced the medical expenditures of individuals treated in general hospitals by 22.2%, and this result was significant at the level of 1%. However, utilizing the Internet sometimes had no effect on the medical costs of individuals treated in general hospitals. Column (4) reports the impact of the frequency of Internet use on the medical expenditures of individuals treated in primary hospitals. The results showed that, under the same conditions, as compared with individuals who never used the Internet, utilizing the Internet almost every day or utilizing the Internet sometimes had no impact on the medical expenditure of individuals treated in primary hospitals, which means that the frequency of Internet use had no impact on the medical expenditure of individuals treated in primary hospitals.

## 4. Robustness Test

To examine the robustness of the results above, we replaced the outcome variables to test the robustness of the results. According to the 2018 CFPS questionnaire design, we chose the question, “Degree of importance of getting information by using the Internet” as the alternative variable for Internet access and frequency of Internet use. The respondents make their choice from 1–5, meaning “very unimportant” to “very important”, respectively. Generally speaking, the higher the importance of using the Internet to access information, the greater the degree of medical information overflow from the Internet.

Table 4 presents the regression analysis results of the impact of the alternative variable on individuals’ medical expenditure. Specifically, the results in Column (1) suggest that the higher the importance of using the Internet to obtain information, the lower the individuals’ medical expenditure (decrease by 1.82%). In Table 4, Columns (2) and (3) divide the hospital levels into general hospitals and primary hospitals, respectively, and further report the impact of the importance of using the Internet to access information on individuals’ medical expenditures among different hospital levels. The results in Columns (2) and (3) suggest that the higher the importance of using the Internet to obtain information, the lower the medical expenditures of individuals treated in general hospitals (decrease by 3.12%) but has no impact on the medical expenditure of individuals treated in primary hospitals. It can be seen that the results in Table 4 are consistent with those in Table 2 and Table 3, which further proves the robustness of the above conclusion.

## 5. Discussion

This paper mainly used the Heckman sample selection model to discuss the relationship between Internet use and individuals’ medical expenditure, which avoided the estimation bias caused by sample deviation, and it is the mainstream model for the current research on health expenditure. According to the relevant literature on the impact of medical expenditure, the variables were strictly controlled to solve the estimation bias caused by the omitted variables and to ensure the credibility of the results. At the same time, the multiplex collinearity analysis of the data specifies that the VIF value of each variable is less than 3 (see Table A1), the maximum value is 2.13, and the average value is 1.34, which is far less than 10. This indicates that there is no multicollinearity relationship among variables, and the design of the model is reasonable. In addition, we used the substitution variable method to test the robustness, which further improved the credibility of the results.

### 5.1. The Overflow of Internet Medical Information Has Reduced Individuals’ Medical Expenditure, and the High Frequency of Internet Use Has a Greater Effect 

The rapid rise of medical expenditure in the field of health care has become a common problem faced by most countries around the world, and the failure of the micro medical service market is an important reason for this rise, which is mainly reflected in excessive medical treatment caused by information asymmetry [1,15,16]. Information asymmetry makes doctors tend to “induce” patients to overconsume medical services and drugs, which is, in turn, influenced by the supply of medical information [31,32]. However, with the advance of Internet technology, searching for health information online is becoming more and more popular among the general population, which could reduce the information asymmetry to some extent. The increasing utilization of the Internet has provided a better opportunity for people to seek health information online regardless of its credibility, accuracy, and reliability [20]. Several studies have found that online health information searching can reduce traditional health care service consumption [27,33,34], and this could further reduce individuals’ health care costs. 

We hold that Internet use can improve or even reduce the information asymmetry between doctors and patients, and thus reduce the level of individuals’ medical expenditure. Compared with offline communication channels, the Internet environment has significant advantages in the dissemination of health information [35]. In the Internet society, individuals can achieve accurate access to medical information through search engines; patients can match disease diagnosis information according to their symptoms and obtain detailed drug treatment programs [8], which helps to increase the individual’s understanding of health and disease information. To some extent, the Internet can eliminate the professional knowledge barriers in doctor-patient communication [29], which can reduce the incentive for doctors to “induce demand” and further decrease the medical expenditure for individuals. Previous studies have confirmed that disease diagnosis and processing information can produce substitutes for hospital diagnosis and treatment services. For instance, through a randomized trial, Osman et al. [36] found that informationalized medical education could reduce the hospitalization rate of asthma patients by 54%. Wagner et al. (2001) reached similar conclusions using natural experiments and found that self-diagnosis books and Internet health information were negatively correlated with the utilization of paediatric services [37]. Meischke et al. (2005) found that older people with high blood pressure and heart disease could effectively reduce morbidity by mastering relevant knowledge of prevention and health care through the use of the Internet [30]. 

These studies support the conclusion of the current study to some extent. The overflow of Internet medical information can affect the use of medical services and further influence the level of individuals’ medical expenditure by reducing the information asymmetry in medical services. Moreover, a high frequency of Internet use has an even greater impact on the reduction effect on individuals’ medical expenditure, which implies that high frequency Internet use has an impact on reducing the information asymmetry in doctor-patient relationships. In other words, the higher the frequency of residents’ Internet use, the richer their information search experience, which generates a greater effect on reducing the information asymmetry in the process of medical services and further reduce the level of individuals’ medical expenditure.

### 5.2. The Impact of Internet Use on Individuals’ Medical Expenditure Varies among Different Levels of Hospitals

This study found that Internet use had different effects on individuals’ medical expenditure among different levels of hospitals. The reduction effect of Internet use on individuals’ medical expenditure is mainly concentrated in general hospitals but has no effect in primary hospitals. On the one hand, primary hospitals generally tend to accept patients with common diseases (e.g., a cold or fever) and chronic diseases (e.g., high blood pressure and hyperlipidaemia), which do not generate excessive medical expenditure, and doctors themselves have no motivation to induce demand. 

Furthermore, the medical conditions and quality of physicians in primary hospitals are far lower than those in general hospitals, and there are no objective conditions to induce demand for doctors, and therefore the use of the Internet has no impact on individuals’ medical expenditure. On the other hand, general hospitals have advanced medical equipment and excellent professional physicians (almost all advanced medical resources are concentrated in general hospitals), but the hospital’s income-generating mechanism is not perfect; therefore, the doctors have the incentive to induce demand and generate excessive medical treatment, resulting in an abnormal rise in medical expenditure. The development of Internet technology enables patients to search for health information and medical-related information efficiently, which can improve the problem of “information asymmetry” between doctors and patients, eliminate the motivation of doctors to induce demand, and reduce the medical expenditure level of individuals to some extent.

Our study had some limitations. First, Internet use can break through barriers to physicians’ professional knowledge and provide quick and easy access to information related to health and medical treatment. However, online knowledge dissemination can have negative effects; false health information can mislead patients’ choices of medical treatment, generate a negative impact on individuals’ health status, and interfere with the research conclusions [38]. Different kinds of information can generate different impacts through related mechanism, which is our further research direction in the future. Second, in terms of the measurement indicators of Internet use, we adopted “Internet use” and “the frequency of Internet use” to measure individuals’ use of the Internet; however, these two indicators may not be accurate enough to measure the impact of Internet use on individuals’ medical expenditure. However, more accurate measurement indicators eluded us due to the CFPS data limits. We plan to further discuss, in detail, the impact of Internet use on residents’ medical expenditure, as soon as possible, if we find more appropriate measurement indicators.

## 6. Conclusions

This paper empirically analysed the impact of Internet use on the medical expenditure of Chinese residents based on 2018 CFPS data. The study found that Internet use can significantly reduce the level of individuals’ medical expenditure, and high frequency Internet use has an even greater impact on the reduction of medical expenditure, but Internet use has different impacts among different levels of hospitals. The impact of Internet use on the reduction of medical expenditure is mainly reflected in general hospitals but has no impact in primary hospitals, which is of great implication to future research. Excessive medical treatment mainly exists in general hospitals, and primary hospitals have no incentive to induce demand. Future research should mainly focus on how to control the rapid rise of medical expenditure in general hospitals, and the use of the Internet should provide a new direction for future research.

First, measures should be taken by the Chinese government to optimize the Internet information environment and promote the construction of "Internet + health care". On the one hand, the government is supposed to strengthen the infrastructure construction and further expand the coverage of the Internet, which can provide a convenient information environment for citizens to obtain health information and improve the accessibility of medical information. On the other hand, the government should strongly support medical institutions in building Internet information platforms and encourage them to provide more Internet medical services such as online consultation, health consultation, communication among patients, and self-health management. 

Second, the government can effectively guide the individual’s use of Internet information overflow media to strengthen the health management awareness, and regulate the operation and maintenance management of an online consultation platform to improve the accessibility of health management information related to disease, and eliminate the information asymmetry problem between doctors and patients as soon as possible.

Third, Internet medical service providers must improve the timely feedback mechanism of online health information consultation. In the whole process of disease prevention, diagnosis, treatment, and rehabilitation, the advantages of Internet health information overflow should be used to provide more channels for access to health information, and further promote individual’s health status without increasing the economic burden.

## Figures and Tables

**Table 1:** Variable definitions and descriptive statistics.

Variables	Definition	Mean	Standard Error
Medical expenditure	Continuous variable, yuan (log)	4.904	3.542
Internet use	Categorical variable, Yes = 1 and No = 0	0.192	0.394
Frequency of Internet use	Dummy variable		
Never	Never = 1, otherwise = 0	0.511	0.500
Sometimes	Sometimes = 1, otherwise = 0	0.402	0.490
Almost everyday	Almost every day = 1, otherwise = 0	0.0863	0.281
Age	Continuous variable, years	48.47	15.83
Age ^2^	Continuous variable, years	2600	1600
Gender	Categorical variable, Male = 1 and Female = 0	0.496	0.500
Marriage status	Dummy variable		
Single	Single = 1, otherwise = 0	0.113	0.317
Married	Married = 1, otherwise = 0	0.818	0.386
Divorced/widowed	Divorced/widowed = 1, otherwise = 0	0.0688	0.253
Family size	Continuous variable	4.181	2.076
*Hukou*	Categorical variable, rural hukou = 1 and urban hukou = 0	0.739	0.439
Religious belief	Categorical variable Yes = 1 and No = 0	0.0317	0.175
Self-rated health	Dummy variable		
Excellent	Excellent = 1, otherwise = 0	0.130	0.337
Very good	Very good = 1, otherwise = 0	0.145	0.352
Good	Good = 1, otherwise = 0	0.415	0.493
Fair	Fair = 1, otherwise = 0	0.137	0.344
Poor	Poor = 1, otherwise = 0	0.173	0.378
Chronic disease	Categorical variable Yes = 1and No = 0	4.299	1.520
Weekly exercise	Continuous variable	4.182	8.906
Smoking	Categorical variable Yes = 1 and No = 0	0.302	0.459
Drinking 3 times or more per week	Categorical variable Yes = 1 and No = 0	0.157	0.364
Education	Dummy variable		
Unschooled	Unschooled = 1, otherwise = 0	0.232	0.422
Primary school	Primary school = 1, otherwise = 0	0.209	0.407
Middle school	Middle school = 1, otherwise = 0	0.292	0.455
High school/technical secondary school	High school/technical secondary school = 1, otherwise = 0	0.142	0.349
Junior college	Junior college = 1, otherwise = 0	0.0693	0.254
University or above	University or above = 1, otherwise = 0	0.0552	0.228
Annual household income	Continuous variable yuan (log)	10.918	1.134
Medical insurance	Dummy variable		
BMIUE ^1^	UEBMI = 1, otherwise = 0	0.175	0.380
BMIURR ^2^	BMIURR = 1, otherwise = 0	0.747	0.435
No medical insurance	No medical insurance = 1, otherwise = 0	0.0784	0.269
Hospital level	Dummy variable		
General hospital	General hospital = 1, otherwise = 0	0.354	0.478
Specialized hospital	Specialized hospital = 1, otherwise = 0	0.0574	0.233
Community health service center/health clinic in town or township	Community health service center/health clinic in town or township = 1, otherwise = 0	0.209	0.406
Community health station/village clinic	Community health station/village clinic = 1, otherwise = 0	0.147	0.354
Private clinic	Private clinic = 1, otherwise = 0	0.232	0.422

Note: ^1^ Basic Medical Insurance for Urban Employees (BMIUE) and ^2^ Basic Medical Insurance for Urban and Rural Residents (BMIURR).

**Table 2:** Impact of Internet use on individuals’ medical expenditure.

Variables	(1)	(2)	(3)	(4)	(5)	(6)
	Expenditure Equation	Expenditure Equation	Expenditure Equation	Expenditure Equation	Expenditure Equation	Expenditure Equation
Internet use	−0.0493	−0.0128	−0.0619 *			
	(0.0408)	(0.0339)	(0.0366)			
Almost everyday				−0.202 **	−0.0539	−0.151 ***
				(0.0800)	(0.0534)	(0.0473)
Sometimes				−0.0450	0.0402	−0.0324
				(0.0380)	(0.0302)	(0.0305)
Age	0.00188	−0.0179 ***	−0.0161 ***	−0.000268	−0.0174 ***	−0.0175 ***
	(0.00735)	(0.00500)	(0.00489)	(0.00781)	(0.00507)	(0.00493)
Age ^2^	0.000180 ***	0.000295 ***	0.000288 ***	0.000193 ***	0.000296 ***	0.000296 ***
	(5.59 × 10^−5^)	(4.78 × 10^−5^)	(4.65 × 10^−5^)	(5.56 × 10^−5^)	(4.77 × 10^−5^)	(4.64 × 10^−5^)
Gender	−0.0833	0.0204	−0.00155	−0.0890	0.0176	−0.00494
	(0.0888)	(0.0310)	(0.0305)	(0.0875)	(0.0311)	(0.0304)
Married	−0.0472	0.00621	−0.0402	−0.0504	−0.00477	−0.0415
	(0.0552)	(0.0469)	(0.0456)	(0.0550)	(0.0471)	(0.0457)
Divorced/widowed	−0.168 **	−0.0791	−0.0976	−0.167 **	−0.0896	−0.0959
	(0.0740)	(0.0621)	(0.0602)	(0.0736)	(0.0622)	(0.0603)
Family size	−0.00121	0.000320	−0.0113 **	−0.00236	0.000322	−0.0117 **
	(0.00781)	(0.00576)	(0.00570)	(0.00761)	(0.00575)	(0.00570)
*Hukou*	−0.229 ***	−0.231 ***	−0.0421	−0.241 ***	−0.229 ***	−0.0404
	(0.0362)	(0.0287)	(0.0315)	(0.0380)	(0.0290)	(0.0315)
Religious belief	−0.0227	−0.0272	−0.00415	−0.0196	−0.0265	−0.00129
	(0.0740)	(0.0618)	(0.0596)	(0.0733)	(0.0617)	(0.0595)
Very good		−0.0953 *	−0.0901		−0.0940 *	−0.0891
		(0.0552)	(0.0549)		(0.0549)	(0.0547)
Good		0.158 **	0.111		0.162 **	0.116 *
		(0.0686)	(0.0714)		(0.0677)	(0.0707)
Fair		0.421 ***	0.351 ***		0.426 ***	0.356 ***
		(0.0859)	(0.0890)		(0.0854)	(0.0884)
Poor		1.029 ***	0.892 ***		1.039 ***	0.899 ***
		(0.108)	(0.115)		(0.107)	(0.115)
Chronic disease		−0.174 ***	−0.139 ***		−0.175 ***	−0.140 ***
		(0.0160)	(0.0173)		(0.0158)	(0.0171)
Frequency of exercise		0.00403 ***	0.00245 **		0.00407 ***	0.00250 **
		(0.00125)	(0.00121)		(0.00125)	(0.00121)
Smoking		−0.119 ***	−0.0872 ***		−0.119 ***	−0.0856 ***
		(0.0310)	(0.0299)		(0.0309)	(0.0299)
Drinking 3 times a week		−0.201 ***	−0.165 ***		−0.201 ***	−0.165 ***
		(0.0348)	(0.0338)		(0.0347)	(0.0338)
Primary school			0.0571 *			0.0586 *
			(0.0327)			(0.0328)
Middle school			0.0704 **			0.0740 **
			(0.0353)			(0.0361)
High school/technical secondary school			−0.0280			−0.0227
			(0.0439)			(0.0450)
Junior college			−0.129 **			−0.114 *
			(0.0604)			(0.0607)
University or above			−0.296 ***			−0.266 ***
			(0.0688)			(0.0689)
Annual household income			0.0779 ***			0.0795 ***
			(0.0111)			(0.0111)
BMIUE			0.0292			0.0383
			(0.0584)			(0.0577)
BMIURR			−0.0491			−0.0456
			(0.0474)			(0.0472)
Specialized hospital			0.285 ***			0.286 ***
			(0.0482)			(0.0481)
Community health service centre/health clinic in town or township			−0.674 ***			−0.675 ***
			(0.0309)			(0.0310)
Community health station/village clinic			−1.099 ***			−1.101 ***
			(0.0358)			(0.0358)
Private clinic			−0.950 ***			−0.951 ***
			(0.0324)			(0.0324)
Province	Control	Control	Control	Control	Control	Control
Constant	8.427 ***	8.879 ***	7.685 ***	8.521 ***	8.842 ***	7.684 ***
	(0.667)	(0.223)	(0.226)	(0.699)	(0.237)	(0.226)
Mills lambda	−1.414 **	−0.652 ***	−0.734 ***	−1.358 **	−0.635 ***	−0.718 ***
	(0.694)	(0.205)	(0.220)	(0.678)	(0.203)	(0.219)
Observations	27,594	27,594	27,594	27,594	27,594	27,594

Notes: (1) Due to space limitations, we did not report the regression results of the selection equation; (2) The benchmark variable for marriage status, self-rated health, education, medical insurance, and hospital level in Columns (1)–(6) was “single”, “excellent”, “unschooled”, “no medical insurance” and “general hospital”, respectively; (3) The benchmark variable for Internet use in Columns (4)–(6) was “never”; (4) due to space limitations, we did not report the dummy variables of province in detail; and (5) *** *p* < 0.01, ** *p* < 0.05, and * *p* < 0.1.

**Table 3:** Impact of Internet use on individuals’ medical expenditure among different hospital levels.

Variables	(1)	(2)	(3)	(4)
	General Hospital	Primary Hospital	General Hospital	Primary Hospital
	Expenditure Equation	Expenditure Equation	Expenditure Equation	Expenditure Equation
Internet use	−0.0963 *	−0.0181		
	(0.0583)	(0.0485)		
Almost everyday			−0.222 ***	−0.0487
			(0.0702)	(0.0691)
Sometimes			−0.00490	0.0385
			(0.0530)	(0.0396)
Age	−0.0154 *	−0.0146 **	−0.0169 **	−0.0160 ***
	(0.00848)	(0.00606)	(0.00858)	(0.00614)
Age ^2^	0.000285 ***	0.000269 ***	0.000297 ***	0.000277 ***
	(7.93 × 10^−5^)	(5.81 × 10^−5^)	(7.95 × 10^−5^)	(5.80 × 10^−5^)
Gender	0.0855 *	−0.0841 **	0.0822 *	−0.0881 **
	(0.0490)	(0.0423)	(0.0491)	(0.0421)
Married	−0.153 **	0.0194	−0.163 **	0.0213
	(0.0780)	(0.0575)	(0.0784)	(0.0578)
Divorced/widowed	−0.227 **	−0.0298	−0.234 **	−0.0254
	(0.105)	(0.0744)	(0.106)	(0.0745)
Family size	−0.0116	−0.00848	−0.0114	−0.00887
	(0.0106)	(0.00685)	(0.0106)	(0.00687)
*Hukou*	−0.0594	−0.0805 *	−0.0557	−0.0817 **
	(0.0524)	(0.0414)	(0.0526)	(0.0414)
Religious belief	0.0217	−0.0302	0.0258	−0.0290
	(0.105)	(0.0732)	(0.105)	(0.0730)
Very good	−0.316 ***	0.0229	−0.321 ***	0.0292
	(0.0998)	(0.0675)	(0.0998)	(0.0669)
Good	−0.192	0.266 ***	−0.196	0.279 ***
	(0.128)	(0.0903)	(0.128)	(0.0885)
Fair	−0.0373	0.545 ***	−0.0445	0.560 ***
	(0.162)	(0.110)	(0.162)	(0.108)
Poor	0.462 **	1.132 ***	0.455 **	1.154 ***
	(0.191)	(0.154)	(0.191)	(0.152)
Chronic disease	−0.0616 **	−0.199 ***	−0.0600 **	−0.203 ***
	(0.0259)	(0.0235)	(0.0259)	(0.0231)
Frequency of exercise	0.00309	0.00186	0.00320	0.00185
	(0.00225)	(0.00143)	(0.00226)	(0.00142)
Smoking	−0.141 ***	−0.0392	−0.137 ***	−0.0390
	(0.0526)	(0.0363)	(0.0528)	(0.0363)
Drinking 3 times a week	−0.110 *	−0.161 ***	−0.107 *	−0.161 ***
	(0.0647)	(0.0391)	(0.0648)	(0.0391)
Primary school	0.180 ***	−0.00821	0.176 ***	−0.00588
	(0.0620)	(0.0385)	(0.0624)	(0.0387)
Middle school	0.165 **	0.0171	0.159 **	0.0224
	(0.0652)	(0.0422)	(0.0665)	(0.0437)
High school/technical secondary school	0.0745	−0.0979 *	0.0641	−0.0898
	(0.0763)	(0.0547)	(0.0778)	(0.0566)
Junior college	−0.158 *	−0.0287	−0.160 *	−0.0169
	(0.0946)	(0.0850)	(0.0947)	(0.0865)
University or above	−0.292 ***	−0.163	−0.274 ***	−0.142
	(0.106)	(0.101)	(0.106)	(0.1000)
Annual household income	0.0764***	0.0855 ***	0.0772 ***	0.0869 ***
	(0.0190)	(0.0138)	(0.0191)	(0.0139)
BMIUE	−0.0666	0.177 **	−0.0606	0.189 **
	(0.0888)	(0.0830)	(0.0886)	(0.0811)
BMIURR	−0.0547	−0.0264	−0.0523	−0.0206
	(0.0785)	(0.0621)	(0.0786)	(0.0616)
Mills lambda	−1.426 ***	−0.241	−1.443 ***	−0.192
	(0.309)	(0.328)	(0.309)	(0.322)
Constant	8.133 ***	6.466 ***	8.190 ***	6.440 ***
	(0.379)	(0.287)	(0.382)	(0.285)
Observations	11,363	16,231	11,363	16,231

Notes: (1) Due to space limitations, we did not report the regression results of the selection equation; (2) The benchmark variable for marriage status, self-rated health, education, and medical insurance in Columns (1)–(4) was “single”, “excellent”, “unschooled”, and “no medical insurance”, respectively; (3) The benchmark variable of the frequency of Internet use in Columns (3)–(4) was “never”; (4) Due to space limitations, the dummy variables of province were not reported; and (5) *** *p* < 0.01, ** *p* < 0.05, and * *p* < 0.1.

**Table 4:** Impact of the importance of using Internet to obtain information on individuals’ medical expenditure among different hospital levels.

Variables	(1)	(2)	(3)
		General Hospitals	Primary Hospitals
	Expenditure Equation	Expenditure Equation	Expenditure Equation
Degree of importance of using Internet to access information	−0.0182 **	−0.0312 **	−0.0134
	(0.00802)	(0.0133)	(0.0100)
Other variables	Control	Control	Control
Province	Control	Control	Control
Mills lambda	−0.683 ***	−1.361 ***	−0.237
	(0.221)	(0.310)	(0.329)
Constant	7.416 ***	7.861 ***	6.227 ***
	(0.214)	(0.345)	(0.275)
Observations	27,568	11,350	16,218

Notes: (1) Due to space limitations, we did not report the regression results of the selection equation; (2) Due to space limitations, the results of the dummy variables of province and other controlled variables were not reported; and (3) *** *p* < 0.01, ** *p* < 0.05.

## Appendix A

**Table A1:** Multiple collinearity test.

Variable	VIF	Variable	VIF	Variable	VIF
Internet use	1.56	*Hukou*	1.44	Drinking	1.16
Age	2.01	Religious belief	1.01	Education level	2.13
Age2	1.08	Self-rated health	1.21	Household income	1.36
Gender	1.7	Chronic disease	1.17	Health insurance	1.02
Married	1.24	Frequency of exercise	1.02	Hospital level	1.13
Family size	1.2	Smoking	1.56	Province	1.08
